# Measuring Road Network Vulnerability with Sensitivity Analysis

**DOI:** 10.1371/journal.pone.0170292

**Published:** 2017-01-26

**Authors:** Leng Jun-qiang, Yang Long-hai, Wei-yi Liu, Lin Zhao

**Affiliations:** 1 School of Automobile Engineering, Harbin Institute of Technology at Weihai, Weihai, P. R. China; 2 School of Transportation Science and Engineering, Harbin Institute of Technology, Nan Gang District, Harbin, P. R. China; Beihang University, CHINA

## Abstract

This paper focuses on the development of a method for road network vulnerability analysis, from the perspective of capacity degradation, which seeks to identify the critical infrastructures in the road network and the operational performance of the whole traffic system. This research involves defining the traffic utility index and modeling vulnerability of road segment, route, OD (Origin Destination) pair and road network. Meanwhile, sensitivity analysis method is utilized to calculate the change of traffic utility index due to capacity degradation. This method, compared to traditional traffic assignment, can improve calculation efficiency and make the application of vulnerability analysis to large actual road network possible. Finally, all the above models and calculation method is applied to actual road network evaluation to verify its efficiency and utility. This approach can be used as a decision-supporting tool for evaluating the performance of road network and identifying critical infrastructures in transportation planning and management, especially in the resource allocation for mitigation and recovery.

## 1 Introduction

As an open complex system, road network is easy to be disturbed by internal or external factors, including energy flow, information flow, material flow and other factors, which make the sub-systems or main parts of road network performance degrade or break down[1,2,3]. In such a recurrence, the levels will be enlarged with more and more corrupted main parts, and finally it may lead to collapse of the whole complex system. This character is called vulnerability. Vulnerability of complex system, as a theory in complex system security research, focuses on the decreasing or breakdown of the complex system’s functions [4,5,6,7]. It is a totally new subject in complex system researches. This theory has seized the eyes of scholars from various fields and has been applied in ecosystem, electric system, network system and other systems.

### 1.1 State of the Art

The existing studies on complex system vulnerability have made certain achievements, putting forward its definition, property, evaluation methods and establishment of a basic theory frame[8,9,10]. Researches on the vulnerability of road network originated in the 1995 Kobe Earthquake in Japan and the 911 event in 2001. These events not only exposed the vulnerability of the interrupted road network, but also showed our deficient researches on the extent and impact of these interruptions [11]. After the 911 event, researches on vulnerability and reliability of road network have attracted a wild attention of scholars [12,13].

The road network vulnerability was proposed by Berdica (2002)[14], which was seen as a susceptibility to incidents that can result in considerable reductions in road network serviceability. Subsequent researchers attempted to indicate the connection between vulnerability and risk, with two components of probability and consequence of vulnerability, e.g. Nicholson and Dalziell and (2003)[15] D’Este and Taylor (2003)[16]. Some scholars used terms like efficiency, capacity and runnability to take place of serviceability. Taylor et al. called the accessibility of nodes, which are reduced by the degradation or deterioration of several road links, the network nodes vulnerability [17]. Chen et al. [11] associated vulnerability of road network with the probability and consequences of risks. Taylor and D' Este[18] thought road network’s vulnerability, reliability and risks are tightly interlinked concepts. Bell et al.[19] analyzed vulnerability of road network through the theory of games. Jenelius et al. [20, 21] considered that the concept of vulnerability should be divided into two sections: One is the probability of actual risks, while the other is the consequences of events. Vulnerability could be dealt with through the risk measurement. Erath et al.[22]defined vulnerability as product between the probability of dangerous situations and the sum of direct and indirect consequences caused by interruption.

The typical methods to evaluate vulnerability include: Scholars like Sohn et al. [23,24] used the road network performance index to study vulnerability and recognize the critical unit of road network, Jenelius et al.[20] with the given OD pairs matrix of road network, made some nodes in network unreachable by constantly removing certain links to analyze road network vulnerability and calculate vulnerability of northern Sweden. Taylor et al.[17] estimated vulnerability of Australian road network with 3 methods, namely generalized travel cost, Hysen integral reachable index and distant degree index. Chen et al.[11] brought in incorporated travel demand model to analyze road network vulnerability. Meanwhile, they took variation of travel demand and supply into consideration and adopted a utility-based reachable index to measure vulnerability of network. Abdul Quium et al.[25] used the γ and α index in graphics to study the connectivity of road network in Bangladesh and analyzed the vulnerability of road network during the 1998 great flood. Ferber et al.[26] performed simulation study on vulnerability of public road network in 14 metropolises by simulating diverse attack strategies and removing different nodes. Murray-Tuite and Mahmassani [27] viewed the identification of vulnerability as the matter of game between “a wicked entity” and road management organizations. To improve computational efficiency, a sensitivity analysis-based method has been proposed by Luathep et al.[28], and this method is likely to be used in large-scale road network vulnerability analysis. It only requires a single calculation with the network equilibrium problem for the proposed method. Meanwhile, the proposed technique remarkably reduces computational burden and memory storage requirements in comparison with the traditional method. Chen et al. [1]proposed an “impact area” vulnerability analysis approach to evaluate the consequences of a link closure within its impact area instead of the whole network. The proposed approach can significantly reduce the search space for determining the most critical links in large-scale networks.

### 1.2 The Objectives and Organization of the Study

While the most studies mentioned above focus on locating the critical infrastructure, pay less attention to the vulnerability of route, OD pair and road network system. It is important for road authorities to know the whole road network operational performance, especially some critical route, because this enables them to protect or to improve those links or parts of the network. There is an urgent need to extend the vulnerability analysis from single road unit to route, OD pair and whole road network. About the computational efficiency, computational burden is still the bottleneck in analysis, especially when the traffic assignment model is repeatedly required in the situation where certain interrupted units need studied successively, it will have heavy computational load, taking lots of computer calculation time, which severely impact its promotion in practice.

In view of the above, this article principally focuses on the establishment of vulnerability model of road network units, route, OD pair and road network and emphasizes that the effective calculation method can be used into real road network. First, the focus will be placed upon the demonstration of traffic utility. Generalized travel time is introduced to reflect the traffic utility on the basis of travel time and reliability. Second, we will define the vulnerability from the perspective of traffic performance sensitivity to disturbances, and establish the vulnerability model of traffic basic unit, path, OD pair and road network on the base of traffic utility. Third, the sensitivity analysis will be adopted to avoid repeated traffic assignment and improve the calculation efficiency. The model will be used in a real road network to verify its applicability, and to identify the critical vulnerable infrastructures of the studied road network. This measure can be used for robust network planning and design.

This paper is organized as follows: the following section defines vulnerability and proposes the vulnerability model. Then, section 3 establishes the vulnerability models for route, OD pair, route and road network. In Section 4, a real example is provided to validate the applicability of the proposed model. Finally, section 5 presents the conclusions.

## 2 Vulnerability of Road Unit

### 2.1 Utility Function

While certain road unit(s) degenerates or diminishes its operational performance, its traffic will transfer to other alternative routes. As a result, the performance of the alternative routes will decrease, and then a domino phenomenon will occur. In other words, this results in the performance of other units, even the whole traffic system decreasing rapidly and the vulnerability arises. The subsequent consequences are the increase of travel time (even some roads are paralyzed and certain nodes are unreachable) and travel cost. Therefore, utility function is introduced to measure the operational performance influence that the degraded road unit brings on other units.

The degeneration of traffic operation is always represented as the increasing of travel time and the decreasing of travel time reliability. Therefore, a concept of generalized travel time is introduced to study the utility of traffic operation. Travel time is to be dealt with in a dimensionless way, for the dimension of travel time is second(s), and there is no dimension for reliability. In this paper, the dimensionless refers to the ratio of vehicles' actual travel time in disturbed traffic flow to free travel time in free flow. Based on this, in this paper, generalized travel time can be defined as the weighted integration between the ratio of actual travel time to free travel time and the unreliability of travel time. It may be written as

$$T_{ga}=\eta (T_{a}/t_{a})+(1-\eta )r_{a}$$ (1)

where *T*_*ga*_ is the generalized travel time on road segment *a*; *η* is the direct proportion coefficient, which shows the weight that the change rate of the travel time occupies in generalized travel time. It could vary for different segments and different people, we can determine this parameter by SP (Stated Preference) Survey. Accordingly, (1 − *η*) shows the weight that the randomness and uncertainty of travel time unreliability occupies in generalized travel time; *t*_*a*_ is the free travel time on road segment *a*; *r*_*a*_ is the probability that road segment *a* does not meet the requirement of reliability; *T*_*a*_ is the travel time on road segment *a*, generally BPR function is used to calculate the travel time on road segment. The formula may be written as

$$T_{a}=t_{a}\left[1+\beta {\left(\frac{x_{a}}{C_{a}}\right)}^{n}\right]$$ (2)

where *x*_*a*_ is the traffic volume on road segment *a* pcu/h; *C*_*a*_ is the capacity of road segment *a* pcu/h; *β*, *n* are the parameters of BPR function, which can be calibrated by field observation data.

Travel time reliability is the probability that travelers carry out their travel plans within a specific time on a certain road network at the specific demand level of service[29]. The formula adopted in this paper is

$$R_{a}=P[T_{a}\leq t_{a}(1+\delta )]$$ (3)

where *δ* is the additional ratio that the travel time accepted by travelers compared with free travel time, *δ*>0. Because the road capacity is a random variable, the travel time is always larger than free travel time. Generally speaking, people have different tolerance against congestions at different locations and it may vary for different travelers. Which can be determined by SP (Stated Preference) Survey.

Put Eq (2) into Eq (3), then

Ra=P[Ca≥xa(δβ)−1n] (4)

As capacity *C*_*a*_ is a random variable, it must obey a certain probability distribution. It usually follows normal distribution. We may get the distribution functions by field observation. Supposing its distribution function is $F_{C_{a}}(x)$, then

Ra=1−FCa(xa(δβ)−1n) (5)

ra=1−Ra=FCa(xa(δβ)−1n) (6)

Since travelers always expect that the shorter routes, the less travel time and the higher reliability, namely, the smaller generalized travel time, the higher traffic utility[30, 31], this paper defines road unit’s utility function as the reciprocal of generalized travel time. The equation may be written as

ea=1η(Ta/ta)+(1−η)ra=1η[1+β(xaCa)]+(1−η)FCa(xa(δβ)−1n) (7)

### 2.2 Sensitivity Analysis for Traffic Utility

The most direct influence of emergencies, such as traffic accidents, road maintenances, bad weather (rain, snow and fog) and terrorist incidents etc., on road traffic operational performance is making capacity decrease sharply, further leading to rapid declining of traffic utility [32]. At present, the common method is re-assigning traffic in the whole road network according to the capacity degradation. However, due to the large computational load of traffic assignment and the long computation time, the computational efficiency is affected. This article adopts sensitivity analysis proposed by Luathep [17], displays the utility index under capacity degradation as Taylor series expansion of the utility under normal conditions and the capacity degradation. The first-order Taylor series approximation may be written as

$$E_{A}{}^{\text{deg}raded}=E_{A}{}^{normal}+\nabla_{C_{a}}E_{A}{}^{normal}{|}_{C_{A0}}.{\left(C_{A}-C_{A0}\right)}^{T}$$ (8)

where $E_{A}{}^{\text{deg}raded}$ is the set of utility index of degraded road network due to a capacity reduction; $E_{A}^{normal}$ is the set of initial utility index of road network under normal conditions; *C*_*A*_ and *C*_*A*0_ are the vectors of road segments capacity in degraded capacity conditions and normal conditions, respectively, *C*_*a*_ ∈ *C*_*A*_, *C*_*a*0_ ∈ *C*_*A*0_; $\nabla_{C_{a}}E_{A}{}^{normal}$ is the Jacobian of $E_{A}^{normal}$ with respect to *C*_*a*_ at *C*_*a*0_, $\nabla_{C_{a}}e_{a}^{normal}\in \nabla_{C_{a}}E_{A}^{normal}$. From Eq (7), it can be concluded that $\nabla_{C_{a}}e_{a}^{normal}$ can be written as

$$\nabla_{C_{a}}e_{a}^{normal}=\frac{\partial e_{a}}{\partial C_{a}}=\frac{\eta n\beta {\left(\frac{x_{a}}{C_{a}}\right)}^{n-1}\frac{x_{a}}{C_{a}{}^{2}}-(1-\eta )\nabla_{C_{a}}F_{C_{a}}(x)\nabla_{C_{a}}x_{a}}{{\left[\eta +\eta \beta {\left(\frac{x_{a}}{C_{a}}\right)}^{n}+(1-\eta )F_{C_{a}}(x_{a}{\left(\delta /\beta \right)}^{-\frac{1}{n}})\right]}^{2}}$$ (9)

### 2.3 Vulnerability Model

In this paper, vulnerability is defined as the sensitive degree of traffic utility when some units (subsystems) or the whole system is disturbed by the internal or external factors. It reflects the sensitivity of traffic operational performance to disturbance. From another viewpoint, vulnerability is the property that how the traffic performance is easily interfered.

This paper uses utility index to show traffic performance, and regards the variation ratio of disturbed utility index to original value as how sensitive traffic performance is to disturbances. The vulnerability function can be expressed as

$$V_{A}=\frac{E_{A}{}^{normal}-E_{A}{}^{\text{deg}raded}}{E_{A}{}^{normal}}$$ (10)

where *V*_*A*_ is the road network unit vulnerability.

The road segment vulnerability can be expressed as

$$V_{a}=\frac{e_{a}{}^{normal}-e_{a}{}^{\text{deg}raded}}{e_{a}{}^{normal}}$$ (11)

where *V*_*a*_ is the vulnerability of road segment *a*, 0 ≤ *V*_*a*_ ≤ 1. Obviously, the smaller *V*_*a*_ is, the smaller the vulnerability is, the more robust it is, more difficult to be disturbed by outside disturbances. Conversely, the larger *V*_*a*_ is, the more vulnerable it is, more highly sensitive to external disturbances; $e_{a}{}^{normal}$ is the initial utility of road segment *a* under normal traffic conditions; $e_{a}{}^{\text{deg}raded}$ is the utility of road segment *a* in a degraded network due to a capacity reduction.

Then, the road segment vulnerability can be expressed as

Va=ηnβ(xaCa)n−1.xaCa2−(1−η).∇CaFCa(xa(δβ)−1n)η+ηβ(xaCa)n+(1−η)FCa(xa(δβ)−1n).ΔCa (12)

## 3 Establishment of Vulnerability Model of Road Network

The series connection of road units constitute route, the parallel connection of one or more routes constitute OD pairs, and hybrid of several OD pairs makes up road network. Therefore, according to different research ranges, vulnerability can be divided into 4 classes, including road segment vulnerability, route vulnerability, OD pair vulnerability and road network vulnerability.

### 3.1 Model for Route Vulnerability

Route vulnerability is the sensitive degree of the route traffic performance when disturbed by internal or external factors. It is closely related to vulnerability of all the components, which constitute routes. Most of the existing researches believe the series connection of road units constitute route and that its relevant evaluation index, e.g. reliability and vulnerability, has two ideas of calculation: one is that the routes’ evaluation index is determined by the worst performance one in all units that constitute routes; the other is to treat it as a general series connection system, in other words, it is to multiply the performance index of all units. The above two ideas of calculation both have irrationality. The first approach is of overgeneralization, over emphasizing the effect of a certain unit in entirety, while the irrationality of the second idea is also obvious. For instance, if the vulnerability of one unit in route is 0 (traffic utility does not decrease), then even though the other units’ vulnerability tends to 1 (traffic utility tends to 0 after disturbance, namely the traffic flow is interrupted and in a state of paralysis), the vulnerability of this route is 0 definitely when multiplying. In other word, the whole traffic flow is not disturbed, which is unpractical.

Due to the irrationality of the above two ideas, the route vulnerability is defined as arithmetic average of all units that make up routes, which can be expressed as

$$V_{k}^{st}=\underset{a\in A}{\sum }\delta_{a,k}^{st}V_{a}/\underset{a\in A}{\sum }\delta_{a,k}^{st}$$ (13)

where $V_{k}^{st}$ is the vulnerability of route *k* between OD pair *s* to *t*; *V*_*a*_ is the vulnerability of road segment *a*; $\delta_{a,k}^{st}$ is 0–1 variable, if road segment *a* is located in route *k* between OD pair *s* to *t*, $\delta_{a,k}^{st}=1$, or $\delta_{a,k}^{st}=0$.

### 3.2 Model for OD Pair Vulnerability

When we define OD pair vulnerability, we have taken the sensitivity of all routes between given OD pairs into consideration. Therefore, OD pair vulnerability is an evaluation index about this OD utility change. The existing researches about OD pair traffic performance evaluation, e.g. reliability, mostly adopt parallel connection theory. There are multiple routes between OD pair *s* to *t*, $V_{k}^{st}$ is the vulnerability of route *k* in OD pair *s* to *t*. Suppose there are *K* routes, OD pair vulnerability can be regarded as parallel connection of all *K* routes, and it may be expressed as

$$V_{st}=1-\overset{K}{\underset{k=1}{\prod }}\left(1-V_{{}^{k}}^{st}\right)$$ (14)

where *K* is the number of routes between OD pair *s* to *t*; *V*_*st*_ is the vulnerability of OD pair *s* to *t*.

This idea also has its irrationality. Assuming there are 10 routes between a given OD pair, and vulnerability of each route is 0.1, according to the above idea, OD pair vulnerability is 0.65. When the number of routes is 30, the result will be 0.958. If the number of routes increases further, the vulnerability would tend to 1 (namely, the traffic is interrupted seriously or paralyzed). Obviously, this result does not reflect the fact, which indicates that if vulnerability of each route is 0.1, the vulnerability of whole OD pair is still 0.1.

In view of the above, we make a breakthrough against traditional parallel connection theory, defining OD pair vulnerability as weighted average of all routes vulnerability between the given OD pair, the weight of each route is defined as the share of the route traffic volume in whole OD pair traffic volume. Then OD pair vulnerability may be expressed as

$$V_{st}=\sum_{k=1}^{K}V_{k}^{st}\cdot q_{{}_{k}}^{st}/\sum_{k=1}^{K}q_{{}_{k}}^{st}$$ (15)

where $q_{{}_{k}}^{st}$ is the traffic volume of route *k* between OD pair *s* to *t*, pcu/h.

### 3.3 Model for Road Network Vulnerability

Road network vulnerability is closely related with the performance of all OD pairs that make up road network. It is a comprehensive index to assess the road network, synthesizing vulnerability of all OD pairs within the road network. Road network vulnerability depends on its OD pairs’ vulnerability. Because trip volumes are different among OD pairs, road network vulnerability can be regarded as the weighted average of all OD pairs’ vulnerability. It may be written as

Vn=∑s=1SVst⋅(qst∑S=1Sqst) (16)

where *S* is the number of OD pairs in the road network; *q*_*st*_ is the traffic volume of OD pair *s* to *t*, pcu/h. *V*_*n*_ is the road network vulnerability.

## 4 Case Studies

**Fig 1:**
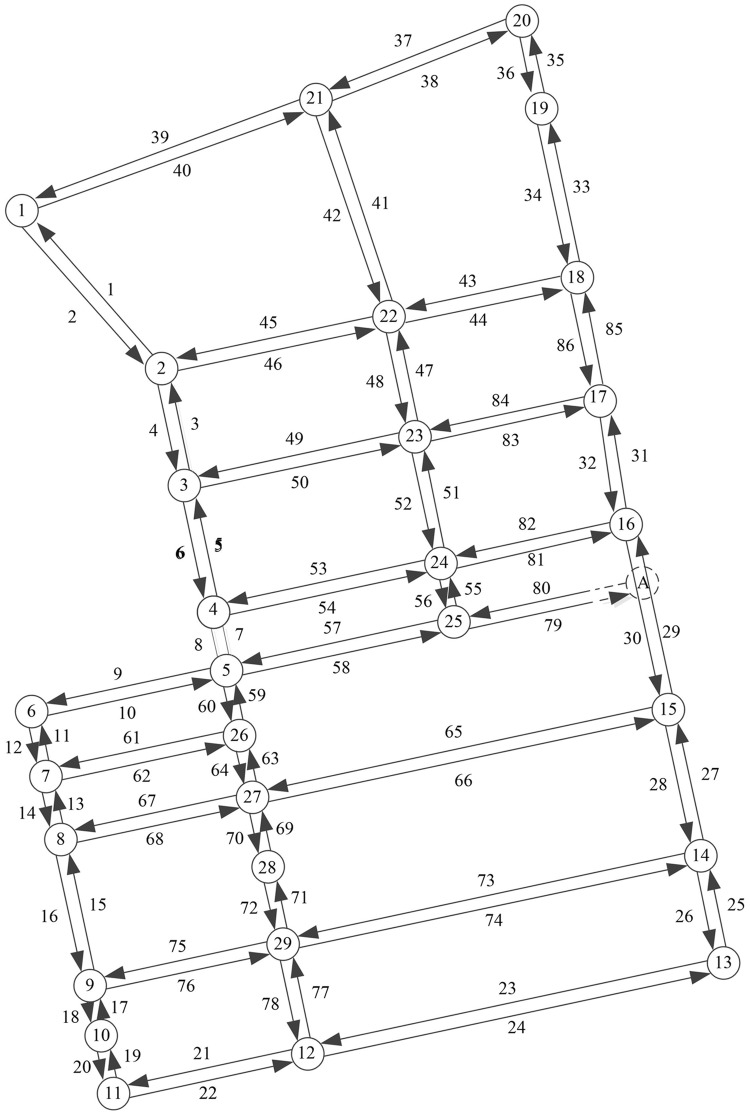
Partial road network in Harbin.

A real medium-seized road network in Nangang district Harbin city of China, as shown in Fig 1, is selected as an example to demonstrate the applicability and efficiency of the proposed method. The road network consists of 84 directed segments and 29 nodes. The segments travel time model is set to *T*_*a*_ = *t*_*a*_[1+0.15(*x*_*a*_/*C*_*a*_)^4^]. The practicality is proved by the evaluating function proposed in this article, such as evaluating traffic utility, studying the vulnerability of each road unit/route/OD pair/road network, and determining the critical components of road network. The efficiency is evaluated in terms of the computational time required of the two approaches (sensitivity analysis and traditional traffic assignment).

Assuming based on normal capacity and capacity reduction by 10%, respectively, the vulnerability of each road segment will be calculated according to Formula (12), thus the critical vulnerable road segments will be conveniently and correctly recognized. In the case study, the critical road segments are 7, 8, 55, 56, 75, 76, 77 and 78, which vulnerability is more than 0.75. According to Formula (13), the vulnerability of each route of all OD pairs will be calculated. Due to the limitation of this paper, only part of the routes in OD pair(1, 13) is listed in Table 1. From the results, it can be seen that the routes 2-4-6-8-60-64-70-72-78-24 and 2-4-6-8-60-64-66-28-26 in OD pair (1, 13) are vulnerable, the vulnerability are 0.626 and 0.536, respectively. Based on Formula (15), the vulnerability of OD(1, 13), OD(20, 11), OD(13, 1) and OD(11, 20) are respectively 0.452, 0.409, 0.603, 0.582. According to Formula (16), the vulnerability of the road network is 0.510.

**Table 1:** Vulnerability of partial routes of OD (1,13).

NO.	Paths	Vulnerability
1	40-38-36-34-86-32-30-28-26	0.372
2	40-42-44-86-32-30-28-26	0.401
3	40-42-48-83-32-30-28-26	0.440
4	40-42-48-52-81-30-28-26	0.414
5	2-46-44-86-32-30-28-26	0.453
6	2-4-50-83-32-30-28-26	0.469
7	2-4-6-8-60-64-70-72-78-24	0.626
8	2-4-6-54-81-30-28-26	0.498
9	2-4-6-8-58-79-30-28-26	0.467
10	2-4-6-8-60-64-66-28-26	0.536

In terms of efficiency, sensitivity analysis adopted in this paper makes the calculation time 1/4 of the traditional calculation method.

## Conclusions

1) This article proposed the sensitivity analysis approach to measure the vulnerability of road networks from the capacity degradation perspective. Sensitivity analysis adopted in this paper makes the calculation time 1/4 of the traditional calculation method (traffic assignment), avoiding the traditional traffic assignment, thus largely improving the computation efficiency and making it possible to analyze the vulnerability of the actual large road network.

2) This article took into consideration of travel time and reliability, defined generalized travel time, regarded the reciprocal of the generalized travel time as the traffic utility, and used the variation rate of the traffic utility to quantify the vulnerability of the traffic. It is a breakthrough against traditional series and parallel connection system theory, building the vulnerability models of route and OD pair based on road segments by means of weighted arithmetic average. It constructed the road network vulnerability model based on the vulnerability of OD pairs and used such a model to accurately and rapidly identify the critical road network components and to evaluate the vulnerability of each road segment, route, OD pair and the whole network, thus providing a theoretic basis for road planning and improvement, especially in the resource allocation for mitigation and recovery.

## Supporting Information

S1 Fig Study partial road network in Harbin. (TIF)

S1 Table Attribute data of segments. (PDF)
