# Supplementary figures and images for: Measuring Road Network Vulnerability with Sensitivity Analysis
Source: PLoS One. 2017 Jan 26;12(1):e0170292. doi: 10.1371/journal.pone.0170292 (PMC5268385; doi:10.1371/journal.pone.0170292)

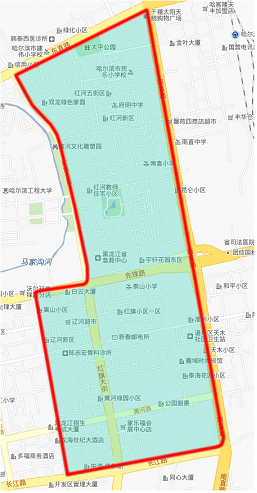

Supplement: S1 Fig — (TIF) [file pone.0170292.s001.tif]
