# Supplementary material for: Measuring Road Network Vulnerability with Sensitivity Analysis
Source: PLoS One. 2017 Jan 26;12(1):e0170292. doi: 10.1371/journal.pone.0170292 (PMC5268385; doi:10.1371/journal.pone.0170292)
Supplement: S1 Table — (PDF) [file pone.0170292.s002.pdf]

Table1 Attribute data of segments

Serials	Name	Grade	O	D	Length/m	Serials	Name	Grade	O	D	Length/m
1	Hongqi St.	Arteries	2	1	810	44	Hongwei Rd.	Branch	22	18	1000
2	Hongqi St.	Arteries	1	2	810	45	Hongwei Rd.	Branch	22	2	560
3	Hongqi St.	Arteries	3	2	550	46	Hongwei Rd.	Branch	2	22	560
4	Hongqi St.	Arteries	2	3	550	47	Hongwei Rd.	Branch	23	22	570
5	Hongqi St.	Arteries	4	3	620	48	Hongwei Rd.	Branch	22	23	570
6	Hongqi St.	Arteries	3	4	620	49	Dayoufang	Branch	23	3	560
7	Hongqi St.	Arteries	5	4	240	50	Dayoufang	Branch	3	23	560
8	Hongqi St.	Arteries	4	5	240	51	Hongwei Rd.	Branch	24	23	540
9	Xianfeng	Arteries	5	6	530	52	Hongwei Rd.	Branch	23	24	540
10	Xianfeng	Arteries	6	5	530	53	Weixing Rd.	Branch	24	4	630
11	Songshan	Secondary trunk	7	6	300	54	Weixing Rd.	Branch	4	24	630
12	Songshan	Secondary trunk	6	7	300	55	Hongwei Rd.	Branch	25	24	550
13	Songshan	Secondary trunk	8	7	290	56	Hongwei Rd.	Branch	24	25	550
14	Songshan	Secondary trunk	7	8	290	57	Xianfeng Rd.	Arteries	25	5	240
15	Songshan	Secondary trunk	9	8	660	58	Xianfeng Rd.	Arteries	5	25	240
16	Songshan	Secondary trunk	8	9	660	59	Hongqi St.	Arteries	26	5	550
17	Songshan	Secondary trunk	10	9	240	60	Hongqi St.	Arteries	5	26	550
18	Songshan	Secondary trunk	9	10	240	61	Liaohe Rd.	Branch	26	7	300
19	Songshan	Secondary trunk	11	10	280	62	Liaohe Rd.	Branch	7	26	300
20	Songshan	Secondary trunk	10	11	280	63	Hongqi St.	Arteries	27	26	540
21	Changjiang	Arteries	12	11	530	64	Hongqi St.	Arteries	26	27	540
22	Changjiang	Arteries	11	12	530	65	Huaihe Rd.	Branch	15	27	300
23	Changjiang	Arteries	13	12	1060	66	Huaihe Rd.	Branch	27	15	300
24	Changjiang	Arteries	12	13	1060	67	Huaihe Rd.	Branch	27	8	1060
25	Nanzhi Rd.	Free Way	14	13	530	68	Huaihe Rd.	Secondary trunk	8	27	1060
26	Nanzhi Rd.	Free Way	13	14	530	69	Hongqi St.	Arteries	28	27	540
27	Nanzhi Rd.	Free Way	15	14	730	70	Hongqi St.	Arteries	27	28	540
28	Nanzhi Rd.	Free Way	14	15	730	71	Hongqi St.	Arteries	29	28	300
29	Nanzhi Rd.	Free Way	15	16	1330	72	Hongqi St.	Arteries	28	29	300
30	Nanzhi Rd.	Free Way	16	15	1330	73	Huanghe Rd.	Secondary trunk	14	29	360
31	Nanzhi Rd.	Free Way	16	17	540	74	Huanghe Rd.	Secondary trunk	29	14	360
32	Nanzhi Rd.	Free Way	17	16	540	75	Huanghe Rd.	Secondary trunk	29	9	540
33	Nanzhi Rd.	Free Way	18	19	720	76	Huanghe Rd.	Secondary trunk	9	29	540
34	Nanzhi Rd.	Free Way	19	18	720	77	Hongqi St.	Arteries	12	29	460
35	Nanzhi Rd.	Free Way	19	20	380	78	Hongqi St.	Arteries	29	12	460
36	Nanzhi Rd.	Free Way	20	19	380	79	Xianfeng Rd.	Arteries	25	A	1000
37	Dongzhi	Arteries	20	21	360	80	Xianfeng Rd.	Arteries	A	25	1000
38	Dongzhi	Arteries	21	20	360	81	Weixing Rd.	Branch	24	16	960
39	Dongzhi	Arteries	21	1	1100	82	Weixing Rd.	Branch	16	24	960

40	Dongzhi	Arteries	1	21	360	83	Dayoufang	Branch	23	17	960
41	Hongwei	Branch	22	21	1100	84	Dayoufang	Branch	17	23	960
42	Hongwei	Branch	21	22	1100	85	Nanzhi Rd.	Free Way	17	18	570
43	Hongtu St.	Branch	18	22	1000	86	Nanzhi Rd.	Free Way	18	17	570
